# Identification of aberrant subvesical bile duct by using intraoperative fluorescent cholangiography: A case report

**DOI:** 10.1016/j.ijscr.2019.07.013

**Published:** 2019-07-19

**Authors:** Toshimitsu Iwasaki, Yoshifumi Takeyama, Yuta Yoshida, Kohei Kawaguchi, Masataka Matsumoto, Takaaki Murase, Keiko Kamei, Atsushi Takebe, Ippei Matsumoto, Takuya Nakai

**Affiliations:** Department of Surgery, Kindai University, Faculty of Medicine, Japan

**Keywords:** IFC, intraoperative fluorescent cholangiography, LC, laparoscopic cholecystectomy, ICG, indocyanine green, MRCP, magnetic resonance cholangiopancreatography, IOC, intraoperative cholangiography, Subvesical bile duct, Ducts of Luschka, Intraoperative fluorescence cholangiography, Cholecystectomy

## Abstract

**Introduction:**

Aberrant subvesical bile ducts are rare structural anomaly located in the peri-hepatic gallbladder fossa. This duct poses the risk for intraoperative bile duct injury resulting in clinically relevant bile leakage.

**Presentation of case:**

Aberrant subvesical bile duct was detected by preoperative magnetic resonance cholangiopancreatography in a 52-year old woman with gallbladder polypoid tumor harboring the risk to be gallbladder cancer. During open cholecystectomy with full thickness dissection, the aberrant duct was identified by intraoperative fluorescent cholangiography (IFC), and dissected safely.

**Discussion:**

Aberrant subvesical bile ducts are mostly found unexpectedly as intra and/or postoperative bile leakage, and remain an important cause of bile duct injuries after laparoscopic cholecystectomy. IFC, which offers real-time imaging of biliary anatomy, has a potential to overcome these problems.

**Conclusion:**

We performed cholecystectomy by using IFC to identify the aberrant subvesical bile duct. To the best of our knowledge, this is the first report showing the fluorescence image of an aberrant subvesical bile duct in a state of nature.

## Highlights

• Aberrant subvesical bile duct poses risk of bile duct injury during cholecystectomy.
• Intraoperative fluorescent cholangiography (IFC) could allow us to identify a fine minute bile duct.
• IFC is one of a promising technique to improve the safety of cholecystectomy further.

## 1 Introduction

Subvesical bile ducts are rare structural anomaly, and, in the most common cases, they are encountered as bile leakage resulting from their injury during cholecystectomy [1]. It is now supposed that bile duct injuries during laparoscopic cholecystectomy (LC) stem principally from misperception of biliary anatomy, not from a consequence of learning curve of surgeon [2]. It is of great significance for surgeon to identify biliary anatomy especially in the patients with its aberrances. Intraoperative fluorescent cholangiography (IFC) is a novel approach with the use of indocyanine green (ICG), which is metabolized by the liver, exerted into bile, and fluoresces by near-infrared light, and therefore, IFC offers real-time imaging of the biliary anatomy [3].

We report our experiences with subvesical aberrant bile duct identified by preoperative magnetic resonance cholangiopancreatography (MRCP) and IFC. The work in this case has been reported in line with the SCARE criteria [4].

## 2 Presentation of case

**Fig. 1:**
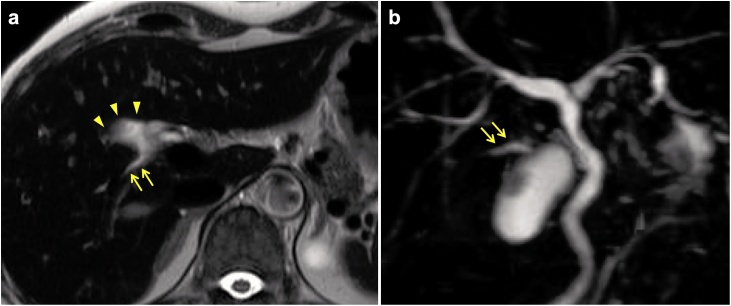
Magnetic resonance image (a) and magnetic resonance cholangiopancreatography (b) showed an aberrant bile duct (arrows) in the gallbladder fossa. Gallbladder was indicated by arrow heads.

**Fig. 2:**
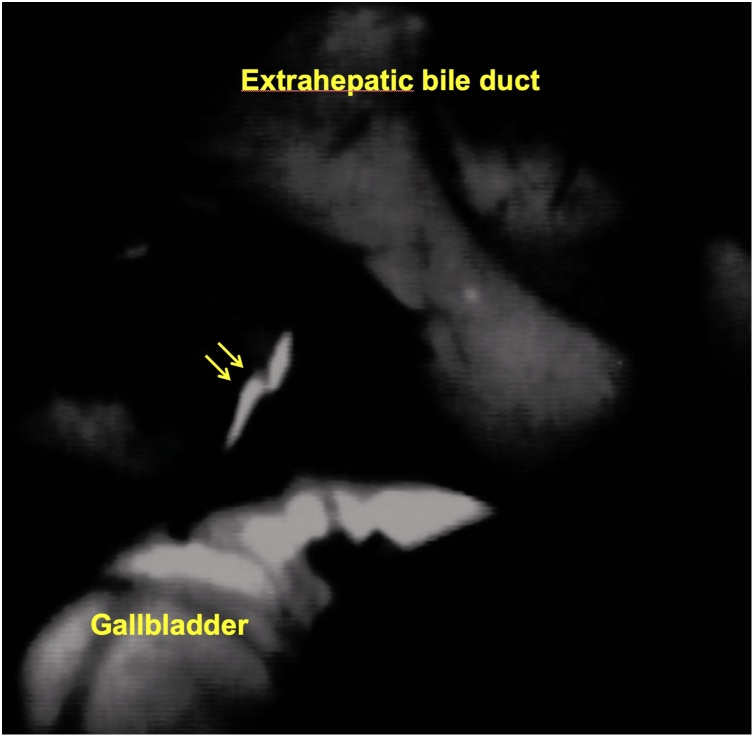
Intraoperative fluorescence cholangiography demonstrated the aberrant bile duct (arrows).

**Fig. 3:**
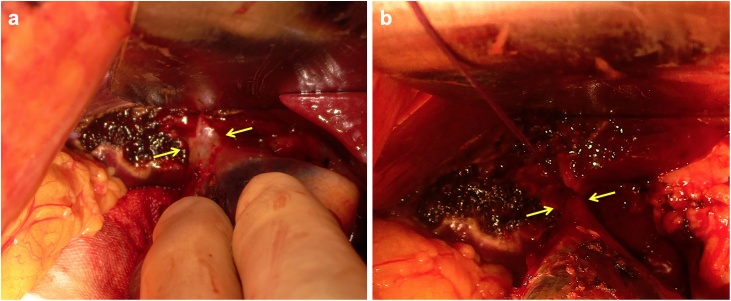
Intraoperative identification (a) and ligation (b) of the aberrant bile duct (arrows) during dissection of gallbladder fossa.

**Fig. 4:**
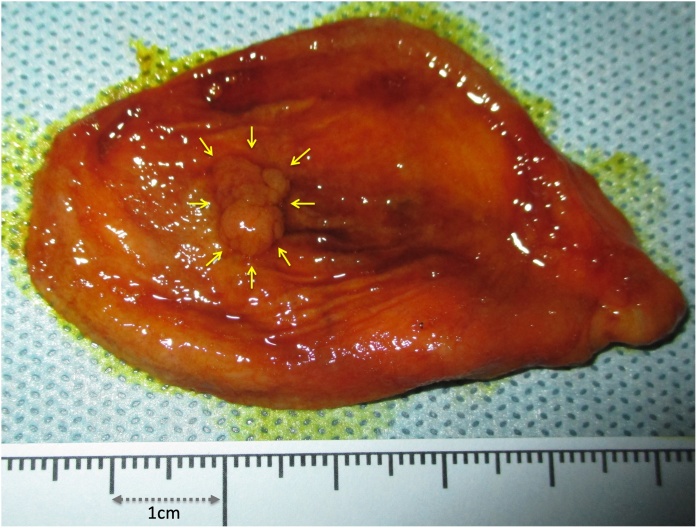
Macroscopic examination of resected gallbladder revealed a papillary polypoid tumor (arrows).

A 52-year old Japanese woman was referred to our hospital for gallbladder polyp. In preoperative work-up, sign of invasion was not apparent, and a string-like structure with high intensity traversing in the peri-hepatic connective tissue of the gallbladder fossa was shown by MRCP suggesting an aberrant bile duct (Fig. 1). Thus, we conducted IFC using during open cholecystectomy with full thickness dissection. Prior to the surgery (just before entering an operation room), 1 mL of ICG (2.5 mg/mL) was intravenously injected. At the time of laparotomy (about one hour after injection of ICG), although the abnormal structure was not identified macroscopically, IFC demonstrated a lumen-like structure with the similar intensity as gallbladder and extrahepatic bile duct (Fig. 2), and the structure was deemed to contain bile juice. Using IFC as a guide, gallbladder fossa was dissected and the duct was identified, ligated and divided (Fig. 3). Communication was not confirmed between the aberrant bile duct and gallbladder by the examination of the specimen, thus, the duct was judged to be aberrant subvesical bile duct (known as “ducts of Luschka”). Macroscopically, a papillary polypoid tumor 11 mm in size was noted in the body of the resected gallbladder (Fig. 4). Based on the pathological examination, the polypoid lesion was a hyperplastic polyp with no-malignancy. The postoperative course was uneventful without bile leakage and the patients was discharged on the postoperative day 7.

## 3 Discussion

Cholecystectomy is one of the most popular surgery, and most of which is performed laparoscopically. Although relatively rare, bile duct injury during LC has been reported with the incidence of approximately 1% or less [[5], [6], [7]], and with their impact on quality of life and survival [8]. Bile duct injury during LC had been supposed to be a consequence of the learning curve of surgeon, but later, it came to be considered that the major cause of bile duct injury during LC was misinterpretation of biliary anatomy [2].

Subvesical bile ducts, which had been termed as “ducts of Luschka”, are rare anomaly of biliary tract traversing in the peri-hepatic connective tissue of the gallbladder fossa [9], and, most commonly, are encountered in clinical practice as a result of their injury during open or laparoscopic cholecystectomy. Several articles carried reports about injuries to subvesical bile ducts [[10], [11], [12], [13], [14]], and Constantine et al. reported that bile leakage from injury of these ducts is the common cause of postcholecystectomy bile leakage. Therefore, surgeons need to acknowledge the possibility of subvesical bile ducts, and preferably, to identify the duct pre-/intraoperatively in a case with this anomaly.

In our case, considering the risk of the patient’s polypoid tumor for malignancy, we made a choice for cholecystectomy with full thickness dissection, which required a dissection to the connective tissue in gallbladder fossa. In addition, preoperative MRCP indicated the prevalence of subvesical bile duct. Therefore, not only to avoid an intraoperative injury of the aberrant bile duct but also to identify and treat the duct safely, we employed IFC in laparotomy. Tsuruda et al. reported the usability of IFC in a case of LC, in which aberrant bile duct was detected unexpectedly by IFC concomitant with angiography after dissecting Calot’s triangle [15]. On the other hand, we performed IFC to identify our patient’s aberrant bile duct intentionally, and the IFC revealed the duct before dissecting the surrounding tissues. These two reports highlighted the usefulness of IFC for identifying the aberrant bile during cholecystectomy.

To reduce the risk of bile duct injury during LC, intraoperative cholangiography (IOC) has been recommended [6,16,17]. Compared to conventional radiographic IOC, IFC has several potential advantages: timesaving, lower risk for bile duct injury associated with　procedure, more convenient technique not requiring the assistance of radiation technician, ease of evaluation for fluorescent images, and no exposure to radiation [18,19]. In addition, fluorescence and imaging can be performed with simple devices (e.g. xenon light source, charge-coupled device camera, laparoscope with lenses that transmit near-infrared light). In the era of LC, IFC should be considered as a means of further improving the safety of this common surgery. On the other hand, IFC has limitation originating in a penetration capability of near-infrared light: 5–10 mm. This technique may be limited in patients with obesity and inflammation due to obstruction of near-infrared light [18].

## 4 Conclusion

We performed cholecystectomy by using IFC to identify her aberrant subvesical bile duct. To the best of our knowledge, this is the first report showing the fluorescence image of an aberrant subvesical bile duct in a state of nature. In the era of LC, IFC is a promising technique to make this common surgery more secure.

## Source of funding

This study did not receive any specific grant from funding agencies in the public, commercial, or not-for-profit sectors.

## Ethical approval

This is case report exempt for ethical approval in our institute.

## Consent

Patient was advised that her clinical data could be used for various studies and comprehensive informed consent was obtained on that basis.

## Author contribution

**Toshimitsu Iwasaki:** conceptualization, methodology, investigation, date curation, writing-original draft, writing-review & editing, visualization.

**Yoshifumi Takeyama:** writing-review & editing, supervision, project administration.

**Yuta Yoshida:** supervision.

**Kohei Kawaguchi:** supervision.

**Masataka Matsumoto:** supervision.

**Takaaki Murase:** supervision.

**Keiko Kamei:** supervision.

**Atsushi Takebe:** supervision.

**Ippei Matsumoto:** supervision.

**Takuya Nakai:** supervision.

## Registration of research studies

Not applicable on this case report.

## Guarantor

Toshimitsu Iwasaki.

## Provenance and peer review

Not commissioned, externally peer-reviewed.

## Declaration of Competing Interest

All authors declare that there is no conflict of interest regarding the publication of this article.
